# A narrative review of norovirus epidemiology, biology, and challenges to vaccine development

**DOI:** 10.1038/s41541-024-00884-2

**Published:** 2024-05-29

**Authors:** Katherine B. Carlson, Anne Dilley, Thomas O’Grady, Jordan A. Johnson, Ben Lopman, Emma Viscidi

**Affiliations:** 1 Moderna, Inc., Cambridge, MA USA; 2 Epidemiologic Research & Methods, LLC, Atlanta, GA USA; 3 Department of Epidemiology, Rollins School of Public Health, Emory University, Atlanta, GA USA

**Keywords:** Diseases, Risk factors

## Abstract

Norovirus is a leading cause of acute gastroenteritis (AGE) globally. AGE resulting from norovirus causes significant morbidity and mortality in countries of all income levels, particularly among young children and older adults. Prevention of norovirus AGE represents a unique challenge as the virus is genetically diverse with multiple genogroups and genotypes cocirculating globally and causing disease in humans. Variants of the GII.4 genotype are typically the most common genotype, and other genotypes cause varying amounts of disease year-to-year, with GII.2, GII.3, and GII.6 most prevalent in recent years. Noroviruses are primarily transmitted via the fecal-oral route and only a very small number of virions are required for infection, which makes outbreaks of norovirus extremely difficult to control when they occur. Settings like long-term care facilities, daycares, and hospitals are at high risk of outbreaks and can have very high attack rates resulting in substantial costs and disease burden. Severe cases of norovirus AGE are most common in vulnerable patient populations, such as infants, the elderly, and immunocompromised individuals, with available treatments limited to rehydration therapies and supportive care. To date, there are no FDA-approved norovirus vaccines; however, several candidates are currently in development. Given the substantial human and economic burden associated with norovirus AGE, a vaccine to prevent morbidity and mortality and protect vulnerable populations could have a significant impact on global public health.

## Introduction

Norovirus is a leading cause of acute gastroenteritis (AGE) worldwide, causing ~685 million cases annually^1^. Each year, an estimated 1.5 million deaths occur globally due to all-cause AGE^2^, with 136,000 to 278,000 related to norovirus infection^2–6^. Young children, older adults, and immunocompromised individuals are particularly vulnerable to severe norovirus disease and its associated complications^2^. In lower-income countries, deaths from norovirus AGE are common among children as a result of severe diarrhea and dehydration^2^; in higher-income countries, deaths occur less frequently and are more commonly observed in the elderly^7^ due to complications from disease including sepsis, cardiac complications, malnutrition, and colon perforation^8^.

## Occurrence of norovirus

### Endemic disease

Estimates of norovirus AGE occurrence differ substantially in publications reflecting variability in methods of surveillance, laboratory test(s) used, study populations examined, and year-to-year variation in circulating genotypes. In one global systematic review of community-based surveillance studies conducted in all ages, norovirus AGE incidence rates ranged widely from 12.5 to 60 per 1000 person-years (PYs)^9^. Surveillance for norovirus in most countries is largely based on outbreak detection with individual cases not reported; therefore, estimates of population-based norovirus AGE incidence come primarily from cohort studies. One cohort study performed among patients enrolled in Kaiser Permanente health plans in the District of Columbia, Maryland, Oregon, and Virginia from 2012 to 2013 estimated the community incidence of norovirus AGE overall as 68.9 per 1000 PYs, and the incidence of norovirus-related outpatient medically attended AGE as 5.6 per 1000 PYs^10^. Norovirus AGE incidence was highest among children <5 years of age (community incidence, 152.1 per 1000 PYs; outpatient incidence, 25.6 per 1000 PYs), followed by older adults aged ≥65 years (community incidence, 75.8 per 1000 PYs; outpatient incidence, 7.9 per 1000 PYs)^10^.

### Outbreaks

Norovirus AGE outbreaks are associated with high attack rates and substantial economic and clinical burden. The most commonly reported setting for norovirus outbreaks in the US and other industrialized countries is healthcare settings. The costs to hospitals associated with outbreaks can be substantial, depending on the number of units affected^11^. Two systematic reviews of norovirus outbreaks occurring in hospitals and nursing homes worldwide found a median attack rate of 50% (range, 9–78) during an outbreak event and a protracted duration of outbreak with a mean of 16 days (range, 3–44) in nursing homes and 19 days (range, 6–92) in hospitals^12,13^. Both patients and staff are important drivers of transmission of norovirus, though evidence suggests a larger number of transmitted infections occur in outbreaks when the index cases are patients than when index cases are staff^14,15^. In a 1-year surveillance study of gastroenteritis outbreaks in three hospitals in England, attack rates of confirmed norovirus AGE during hospital outbreaks were 24.5% for staff (95% CI, 17.8–31.2) and 53.2% for patients (95% CI, 41.5-65.0)^16^. Guidelines to help control outbreaks in healthcare settings include enhanced hand hygiene and environmental cleaning, restriction of patient movements, and exclusion of ill staff from work^17^.

Outbreaks in long-term care facilities (LTCFs) are of particular concern, as individuals receiving care in these settings are more likely to be elderly or have underlying medical conditions. Patients typically live in these facilities for longer than a hospital stay, with daily nursing support and shared rooms and common areas, which increases the potential for rapid spread and larger outbreaks^18–20^. Although person-to-person is the most common transmission route in LTCFs, shared dining facilities may increase foodborne exposure risk^18^. Norovirus AGE attack rates during outbreaks in LTCFs can be up to 45% and are associated with hospitalization rates of ~4% and mortality rates of ~2%^18,21^, making LTCFs an important target for surveillance and control of norovirus.

Other commonly reported settings for norovirus AGE outbreaks include restaurants and catered events, schools and childcare centers, and settings where individuals reside in close contact, such as cruise ships or dormitories^22^. An analysis of norovirus outbreaks reported to the Centers for Disease Control and Prevention (CDC) CaliciNet in the US between 2009 and 2013 reported the most common settings as long-term care facilities (62.5% of outbreaks), restaurants (9.8%), schools and communities (5.7%), parties or events (5.4%), and hospitals (3.6%)^23^. Cruise ship outbreaks are estimated to account for only a small proportion of outbreaks, an estimated 1% in the US between 2009 and 2012; however, the numbers of individual cases associated with these outbreaks can be very large^24^. Since 2006, ~90% of cruise ship outbreaks with known causative agents involved noroviruses^25^. Based on data from the CDC Maritime Illness Database and Reporting System, the incidence rate of AGE on passenger ships was 16.9 cases per 100,000 travel days in 2019^26^. Among passengers, AGE incidence rates increased with increasing ship size and voyage length^26^.

Norovirus AGE causes substantial economic and clinical burden, with direct healthcare costs and lost productivity from personal illness or time spent caring for an ill child estimated to cost $60 billion globally each year^27^. A simulation model estimated the economic burden of norovirus stratified globally and by World Health Organization region to examine direct costs of illness (e.g., clinic visits and hospitalization) and productivity losses due to norovirus^28^. The model estimated annual global costs related to norovirus as $4.2 billion in direct health system costs and $60.3 billion in societal cost^28^. Children aged <5 years account for the highest estimated societal cost collectively while costs per illness are highest for adults aged >55 years^28^. The 2019 Global Burden of Disease Study estimated that children <5 years account for 33% of all deaths and 56% of disability-adjusted life-years (DALYs) globally, with adults aged ≥70 years accounting for 40% of norovirus deaths globally and 11% of DALYs^2^.

A reduction in norovirus AGE incidence was observed during periods of shutdown for COVID-19 ^29,30,31,32^; however, recent data have shown that, with the lifting of COVID-19 pandemic restrictions, norovirus AGE incidence has returned to pre-pandemic levels^33^. Due to limitations in surveillance (based on outbreaks only), it is challenging to determine the full burden of norovirus. Observational studies have attempted to quantify incidence rates in smaller, defined populations, but wider, routine population-based surveillance is needed to fully appreciate the societal impact of norovirus AGE. Real-world studies, specifically those that utilize epidemiologic modeling, may be of utility in assessing norovirus occurrence and the potential impact of vaccines to reduce disease burden.

## Norovirus biology and molecular epidemiology

Noroviruses are icosahedral viruses in the family *Caliciviridae*, with a single-stranded, positive-sense RNA genome^34,35^. Virions are non-enveloped and quite small, about 40 nm at their largest diameter^34^. The genome is a single RNA segment ~7.5 kilobases long, divided into four open reading frames (ORFs)^34^. ORF1 encodes six non-structural proteins, including the RNA-dependent RNA polymerase or RdRp protein^34^. Other ORF1 proteins include an N-terminal protein, a NTPase, a “3A-like protein”, a VPg, and a viral protease^34^. ORF2 encodes the major capsid protein, VP1, which is further subdivided into shell (S) or protruding (P1 and P2) domains. ORF3 encodes the minor capsid protein, VP2^34^. ORF4 is encoded by the murine norovirus subgenomic RNA, in an alternative reading frame overlapping the VP1 coding region^36^.

Noroviruses are genetically diverse and can infect a wide variety of hosts, including humans, dogs, pigs, mice, bats, and sea lions^37,38^. They are divided into 10 genogroups (GI to GX) based on VP1 amino acid sequence^39,40^. Each genogroup is subdivided into genotypes^37–40^ based on capsid amino acid sequence, with 49 genotypes currently described^39^. Noroviruses can also be classified based on their RdRp (“polymerase”) sequence, with at least 60 P-types currently circulating. Each virus strain can be classified based on its capsid and polymerase genotypes, which is similar to the dual-numbering system seen in influenza or rotavirus strains. For example, GII.4[P4] has a GII.4 capsid and a GII.P4 polymerase. Recombination is possible between different polymerase and capsid genotypes, most commonly at the ORF1-ORF2 juncture, leading to many different strains such as GII.13[P16] or GII.3[P12]^39,41–43^. Genogroups GI, GII, GIV, GVIII, and GIX can all infect humans; however, GI and GII genotypes are by far most common, accounting for ~90% of all reported cases and outbreaks in humans^38,39,44,45^.

A specific GII norovirus genotype, GII.4, is most prevalent in human norovirus AGE cases^44,45^. GII.4 viruses have been responsible for the majority of outbreaks and sporadic cases for at least the last 15 years^44,45^. Less is known about norovirus diversity prior to around 2000, when more robust surveillance efforts were established, but earlier large-scale outbreaks in the 1980s and 1990s are known to also have been caused by GII.4 variants, suggesting some consistency with more modern observations^46,47^. It has since been demonstrated that GI and other GII genotypes are relatively genetically static^37,48,49^, while new GII.4 variants have replaced previous ones historically every 2–5 years prior to 2012^37,48,49^. New variants are named when they have become epidemic in at least two geographically diverse locations^39^. Since the mid-1990s, there have been six new GII.4 variants that have caused widespread epidemics: 1996 Grimsby, 2002 Farmington Hills, 2004 Hunter, 2006 Den Haag, 2009 New Orleans, and 2012 Sydney^37^. GII.4 2012 Sydney has persisted for more than a decade now, though it did recombine with a novel polymerase gene around 2015, identified in surveillance as GII.4 Sydney[P16]^50^. The predominance of GII.4 Sydney over the past decade has been hypothesized to be in part due to a lack of immunity in adults, facilitating continued circulation and predominance of this variant^51^.

**Fig. 1:**
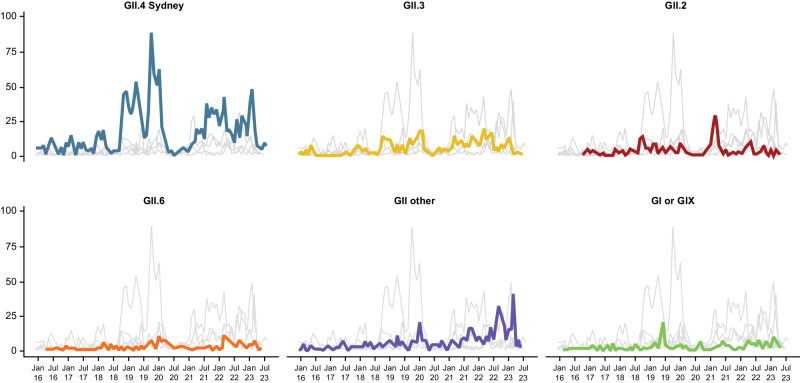
Number of sequences by specimen collection date for the top 4 most prevalent norovirus genotypes and GII other and GI genotypes between 2016 and 2023, among children under 5^56^. Reproduced with permission from NoroSurv. https://www.norosurv.org/login. Accessed December 15, 2023.

Though generally accepted as the most predominant genotype, the exact proportion of disease attributed to GII.4 varies somewhat by setting, surveillance system, geography, year of data collection, and population. For example, prevalence was reported as 58% among outbreaks reported from September 2013 to August 2016 in the United States^52^, 65% in international laboratory-based outbreak surveillance of norovirus specimens from Europe, Asia, Oceana, and Africa collected from January 2005 to November 2016^53^, 67% in published observational studies (2004–2012) of non–outbreak-associated cases in children^54^, and 41% among a birth cohort prospectively followed for the first 2 years of life in Lima, Peru, from June 2007 to April 2011^55^. The next most common genotypes reported in outbreak surveillance from high- and middle-income countries in recent years include GII.2, GII.3, GII.6, and (transiently, primarily in Asia) GII.17^52,53^. Among GI genotypes, GI.3 has been the most common in recent years^52,54^. The exact proportions of disease due to any of these genotypes varies year to year but are almost always less than the proportion attributed to GII.4^53^. Figure 1 presents contemporary genotype data from pediatric norovirus AGE cases collected globally through NoroSurv between 2016 and 2023^56^. The most commonly identified genotypes over the time period were GII.4, GII.3, GII.2, and GII.6.

Studies that have examined norovirus outbreaks over time have observed increases in incidence that correlate with the emergence of new variants. For example, in an analysis of norovirus outbreak data in the US from the National Outbreak Reporting System and CaliciNet from August 2009 to July 2019, the longest norovirus season was observed in 2015–2016, which coincided with the emergence of the GII.4 Sydney[P16] variant, although the peak-to-mean ratio and number of reported norovirus outbreaks were generally similar to other surveillance years^57^. Increases in norovirus incidence have been shown in multiple countries with the emergence of GII.2[P16] in 2015–2016 and GII.17[P17] in 2014–2015. For example, data from norovirus outbreaks in China from 2016 to 2018 found that GII.2[P16] caused an increase in norovirus outbreaks during winter 2016–17^58^. Previous data from China had reported a peak in GII.17 norovirus cases during 2014–2015, followed by a decrease in GII.17 in 2015–2016^59^. An increase in norovirus cases was also observed in Germany in 2016 in association with the emergence of GII.2[P16]^60^. In an epidemiological study in Ibaraki Prefecture, Japan, of surveillance data from September 2012 to August 2018, variation in norovirus frequency over time was observed with the emergence of new variants^61^. However, it is important to note that not all new variant emergences have been associated with increased disease activity, which suggests a complex interplay of viral fitness and immune escape^62^.

Emergent norovirus variants do not appear to originate in an animal reservoir and there is no evidence of zoonotic transmission^63^, which raises the question as to whether there is a human reservoir of these viruses. While none has been clearly identified, molecular epidemiological surveillance is very sparse in lower-middle-income and low-income countries where vast numbers of infections occur^64^ and intra-host viral evolution could contribute to new mutations^65^. It has also been hypothesized that immunocompromised individuals may be a source of new variants^63^.

Evidence suggests that GII.4 infections tend to result in more severe clinical disease than other genotypes^66,67^; however, the question of disease severity has been difficult to study because of many confounding issues. GII.4 viruses appear to be more common among the elderly, especially in LTCF outbreaks, and are often linked to person-to-person transmission. Meta-analyses and large outbreak studies have found evidence for increased severity of disease caused by GII.4 viruses^68^. In one study of infected children, GII.4 cases presented to healthcare providers were more likely to receive oral rehydration fluids, a measure of disease severity, when compared with non-GII.4 cases^69^. This pattern was also reported in a cohort of Canadian children^66^, a study of infected children in Finland^70^, and a study among residents of nursing homes in the Netherlands^71^. In outbreak settings, GII.4 has been shown to be associated with higher hospitalization and mortality rates^67,68^. There are a few possible biologic reasons for higher severity in GII.4 cases, including higher viral load, increased ligand binding, or rapid evolution that evades the host response^68,71–74^. However, it should also be noted that outbreaks caused by GII.4 often occur in healthcare facilities, where populations may be at higher risk of severe disease, which may bias outcomes^68,71–75^.

GII.4 is the most commonly reported genotype across all ages, occurring at similar frequency among children and adults. Importantly, when novel GII.4 variants emerge, they disproportionately cause disease in older age groups^68^. Potential biases arising from the design of surveillance systems should be considered when looking at reported genotype prevalence across cases. In the United States, for example, surveillance is conducted for outbreaks (not individual cases), which are more likely to occur in healthcare settings^75^. Because elderly people are over-represented in healthcare settings, and because GII.4 causes outbreaks (as opposed to “sporadic” cases) more often than other genotypes, this may result in the observed higher burden of GII.4 in older adults.

A 1974 human challenge study with different (heterologous) norovirus genotypes indicated that infection with one genotype is not protective against infection with other genotypes^76^. However, challenge study doses are not thought to reflect natural infection since the infectious doses were unnaturally high and some of the challenge strains are uncommon; as such, results from those studies should be interpreted with caution. Some observational studies of norovirus AGE have similarly demonstrated that infection with one genotype does not protect against subsequent genotypes from the same genogroup^77–79^, while other observational cohort studies have shown protection against both repeated infections with the same genotype (homologous) as well as heterologous genotypes^80^. More recent data from a field efficacy trial conducted in 2016–2018 suggested protection against GII.2 illness after administration of a bivalent GI.1 and GII.4 vaccine^81^. To explain these observed findings, it has been hypothesized that ‘immunotypes’ of groupings of genetically similar noroviruses exist that provide heterotypic immunity within each type^48^. Prevalence of immunotypes may be due to differences in evolution patterns between these types, with genetically static genotypes more prevalent among younger age groups. In contrast, GII.4 may predominate among older age groups because of its high rate of evolution in VP1. This is still an active area of research, with circumstantial evidence supporting real-world cross-protection within immunotypes. Identifying cross-protection at the genotype level in the real world is important but will be challenging since individuals have complex exposure histories and circulating genotypes are ever-changing.

## Transmission and natural history

Transmission of norovirus mainly occurs via the fecal-oral route. Norovirus is highly contagious – though its precise infectiousness remains an area of uncertainty. Based on analysis that considered the aggregation of particles (as visualized by electron microscopy), the infectious dose of norovirus was estimated to be as low as 18 virions^82^. Other estimates that rely on quantified dose ranges given in human challenge studies arrive at higher estimates on the order of 1000 virus particles^83^. Regardless of the true precise estimate, norovirus is a highly infectious agent. The virus spreads directly from person to person or indirectly through contaminated food or water^84^. Infected individuals shed billions of viral particles per gram of stool or vomit, which can contaminate food, water, and surfaces^40^. Vomiting can result in significant environmental contamination^85^, leading to transmission through fomites and airborne droplets. A study of norovirus transmission in LTCFs found that vomiting was a primary driver of transmission^86^. Even after symptoms resolve, transmission can occur. In immunocompetent individuals, viral RNA can be detected in stool for several weeks after symptoms resolve^87,88^; in immunocompromised individuals, chronic illness and viral shedding can persist for weeks to years and become a chronic infection^89–91^. Asymptomatic individuals can also excrete a substantial amount of virus, and therefore are also an important source of transmission. The norovirus viral replication cycle is not fully defined, and the primary human receptor has yet to be identified^92,93^. Current evidence suggests that norovirus viral entry occurs through attachment to histo-blood group antigens (HBGAs) on the surfaces of gut epithelial cells. The presence of HBGAs on host-mucosal surfaces is determined by the fucosyl-transferase-2 (*FUT2*) gene^94,95^. Individuals with functional *FUT2* possess HBGAs necessary for viral docking (“secretors”), whereas individuals with defects in *FUT2* do not express the appropriate HBGA necessary for viral docking (“non-secretors”)^96^. Non-secretors are resistant to some genotypes, including GII.4, GI.1, and some other “secretor-dependent” genotypes^96^. Individuals who are non-secretors are found in ~20% of European populations, with higher prevalence observed in South Asia and lower prevalence observed in Latin America^97^. The relationship between transmission and secretor status may even be strain-dependent, as GII.4 infections have been documented among non-secretors^98^.

Norovirus seasonality can vary according to climate. Temperate climates see norovirus infections and disease year-round, but epidemic peaks are concentrated in the winter^99^, with 63% to 73% of cases occurring during winter months (e.g., October-March in the northern hemisphere)^7,69^. In these climates, norovirus may spread more easily during the winter due to its ability to thrive in colder temperatures^40^ and increased human contact indoors^100^. In tropical climates and the southern hemisphere, norovirus AGE follows a less distinct seasonal pattern, with peaks observed in the winter and cooler and/or rainier months (roughly April to September), but a distribution of cases is also observed throughout the year^101^.

Transmission risk factors include contact with contaminated food, water, and surfaces^18,102,103^. Food supply contamination with norovirus can occur at production or during food preparation^104,105^. High-risk foods include produce (particularly leafy greens and fresh fruits) and shellfish (e.g., oysters)^106–110^. Cooked food later handled by an individual with a norovirus AGE infection may also become contaminated; thus, good hygiene in foodservice establishments is essential to avoid such contamination^13^. Water supply contamination with fecal material containing norovirus can rapidly affect large populations^111–113^. In high-income countries, more infections are transmitted from person to person rather than through exposure to contaminated food or water^96^. Foodborne transmission of norovirus accounts for ~14% of norovirus AGE outbreaks globally^114^; however, data are overwhelmingly from high-income countries. Foodborne and waterborne transmission rate estimates from low-income countries are lacking but are thought to be much higher^96^. Environmental transmission risk factors include cohabitating with large numbers of individuals, contact with infectious individuals, and improper hand washing. Outbreaks occur commonly in settings where people are in close contact, such as dormitories, military centers, prisons, resorts, cruise ships, daycares, and LTCFs. In high-income countries, LTCFs and hospitals are the most common settings of norovirus AGE outbreaks^115,116^. In the United States, 52% of reported norovirus AGE outbreaks occur in LTCFs and 3% occur in hospitals or acute care facilities^115^. However, in Europe, Australia, Canada, and Japan, outbreaks in LTCFs and acute care settings/hospitals are roughly equal in proportion^116^.

## Adaptive immunity

Immunity to norovirus is not well understood but is thought to be imperfect and of limited duration. Recent estimates of the immunity duration to norovirus vary widely, from as little as 27 months to as long as 9 years^117,118^ In earlier human challenge studies, it appeared that immunity following challenge was of short duration (from about 2 months to 2 years)^76,119,120^. It was also observed in some of these studies that there was a subgroup who were entirely resistant to infection or disease, at least from the GI.1 challenge strain^119,120^. It is now known that this resistance is moderated by the FUT2 gene as described above. Understanding of immune acquisition was limited from these studies since they were conducted on adults who already had a lifetime of exposure and used unnaturally large challenge doses of virus^76,117,119,120^. Contemporary birth cohort studies have further advanced understanding of acquired immunity. The MAL-ED study, conducted among birth cohorts in several low- and middle-income country settings, indicated that natural GII subgroup infection provides protection against subsequent gastroenteritis caused by GII and that immunity builds up over multiple infections^121^. However, there was little immunity acquired against infection and no evidence of protection from GI infection^121^. Modeling studies estimate a longer duration of immunity of ~4–9 years to all noroviruses (i.e., not genotype-specific)^118^. As noted in the molecular epidemiology section above, there is no evidence of protection across genogroups and limited cross immunity among genotypes in the same group. HBGA blocking antibody is thought to be associated with protection from norovirus disease and vaccine response^122–126^, but a cutoff value for antibody titer correlated with protection has yet not been defined. Loss of immunity may be a result of antibody and cellular immune memory decay and/or immune escape of emerging genotypes and variants^122,123^.

## Symptoms

Norovirus AGE symptoms usually emerge ~ 1 day after exposure, although a small number of cases exhibit symptoms in as few as 0.5 days^113,127^. Common symptoms include nausea, vomiting, and diarrhea; less common symptoms include fever, lethargy, weakness, and headache^113,128^. Symptoms usually last for ~ 2 days^129,130^. While people with norovirus AGE typically recover quickly, viral shedding can persist for weeks after infection^131^. Norovirus AGE ranges in severity from mild to life-threatening, with young children, elderly, and immunocompromised individuals at the highest risk for severe disease. Most clinical case definitions for AGE require diarrhea; however, a significant portion of norovirus-infected individuals experience vomiting in the absence of diarrhea. One study found that 35% of children aged < 2 years experienced vomiting but not diarrhea^132^. In outbreak investigations, which do not evaluate endemic disease, suspected norovirus is frequently defined as persons experiencing vomiting and/or diarrhea ( ≥ 3 loose stools in a 24 h period) whose symptoms have no other known cause^133^. Studies that require diarrhea in the case definition may underestimate true burden if they do not include vomiting-only illnesses.

Case definitions for norovirus AGE vary by location and institution. According to the US Centers for Disease Control and Prevention, the case definition of norovirus AGE is a disease primarily consisting of vomiting, abdominal cramps, nausea, and diarrhea, with an onset of symptoms 12–48 h after exposure. In Ireland, the definition of norovirus AGE is any person with vomiting (particularly if projectile) and/or diarrhea; detection of norovirus in feces using ≥1 of four laboratory tests (norovirus via electron microscopy; virus-specific RNA; virus-specific antigen; or small round structured virus via electron microscopy); and an epidemiologic link, either human to human transmission or exposure to a common source^134^.

## Diagnosis

Norovirus may be suspected based on symptoms, but routine testing is not usually conducted outside of outbreak investigations. Laboratory confirmation of norovirus is generally not necessary in clinical settings, although it may be useful in select situations, for example in immunocompromised patients with severe or persistent symptoms or for public health purposes during outbreaks of gastroenteritis. The most widely used method is reverse-transcription real-time polymerase chain reaction (RT-PCR) assays, which provides high sensitivity and specificity, can estimate viral load in stool samples^52,105,135^, and can discriminate between pathogens^52,105,135^. Enzyme immunoassays (EIAs) can also be used to diagnose norovirus AGE in stool samples but have poor sensitivity^105,135–138^. Ideally, stool specimens should be collected < 2–3 days from symptom onset and frozen or refrigerated to ensure nucleic acid integrity^135^.

Clinical and epidemiologic criteria are commonly used to identify outbreak cases. Outbreaks of AGE can be attributed to norovirus when there is a mean (or median) illness duration of 12–60 h; a mean (or median) incubation period of 24–48 h; vomiting in >50% of individuals; and no enteric bacteria found^139^.

## Treatment and prevention

### Treatment

To date, no US Food and Drug Administration-approved therapies are available for norovirus AGE^140,141^. Most norovirus AGE episodes last 2–3 days, are self-limiting, and are managed with hydration and supportive care^142^. Severe cases may require medical intervention to alleviate fluid loss, including hospitalization^142^. In lower-income countries with limited access to rehydration therapies, prevention of norovirus AGE is essential. Beyond infection control precautions, rehydration treatments do not differ substantially from the treatment of other non-bacterial causes of diarrhea^27^.

In immunocompromised individuals with persistent norovirus infections^89,91,143,144^, complications can be treated using intravenous fluids, parenteral nutrition, and adjustment of immunosuppression^142^. Given the risk for chronic and severe disease, immunocompromised individuals could potentially benefit from prevention through vaccination or norovirus antiviral treatments^140–142^.

### Vaccine prospects

Norovirus vaccine development is challenging due to the virus’s genetic diversity, lack of a robust cell culture system for in vitro assays, and an incomplete understanding of natural immunity^145^. At the time of this writing, known vaccine candidates in discovery or development are based on virus-like particles (VLPs), mRNA, adenovirus vectors, or P-particles, which use only the P domain of the VP1 protein^145–149^. A combined rotavirus-norovirus vaccine, which includes antigens to both causes of AGE, has also been explored^150–152^.

The most advanced vaccine candidate to date (formerly known as TAK-214 [Takeda Pharmaceuticals]; now being developed by Hillevax as HIL-214) is an intramuscular VLP-based bivalent vaccine that contains antigens to GI.1 and GII.4 genotypes that has been studied in both adult and pediatric populations^153–155^. In a phase 1/2 trial, serum antibody responses to HIL-214 were observed in adult participants, though there was little increase in antibody levels following a second intramuscular dose^154^. A recent phase 2 trial in children aged 6 months and 4 years reported substantial immunogenicity (determined by pan-Ig and HBGA titers) 28 days after HIL-214 dosing, with stabilization or slight increase in titers 28 days after the second dose^155^. A phase 2b field efficacy study of TAK-214 demonstrated higher measures of vaccine-induced immunity (measured through pan-Ig, IgA, and HBGA-blocking antibody titers) than baseline levels, waning 1 year following vaccination. The findings also suggested some cross-genotype protection against non–vaccine-type GII.2 illnesses in vaccinated individuals^81^; further research into the observed cross-protection is warranted. HIL-214 is currently being examined in a phase 2b study in infants, which was initiated in 2022 (NCT05281094).

An oral norovirus vaccine candidate (Vaxart, Inc.) uses a nonreplicating adenovirus-based vector expressing the VP1 gene from the GI.1 norovirus strain, a double-stranded RNA adjuvant, and a bivalent GI.1/GII.4 composition^156^. This vaccine was well-tolerated and led to robust IgA responses in recipients from a single-site, randomized, double-blind, placebo-controlled, phase 1 study^156^. An intramuscular, VLP-based, mRNA vaccine against norovirus is being developed by Moderna, which is currently under investigation in a phase 1/2 study in adults^149^. Two additional vaccines are under clinical investigation in China^147^. The first is a bivalent VLP-based vaccine (National Vaccine and Serum Institute) composed of two recombinant VLPs representing the GI.1 and the GII.4 genotypes^147^. The second is a quadrivalent vaccine consisting of four aluminum salt adjuvanted VP1 proteins representing GI.1, GII.3, GII.4, and GII.17 genotypes^147^. Additional preclinical-stage vaccines are also in development^145,146^.

## Conclusions

Norovirus is now the leading global cause of AGE in many regions in the era of pediatric rotavirus vaccination. Disease burden is highest in young children and older adults, resulting in substantial health and economic impact in both lower- and higher-income countries, including >200,000 deaths a year across all ages and ~70,000 among children under 5. The high transmissibility of the virus and short incubation period make norovirus very difficult to control, leading to outbreaks of substantial cost and size, particularly in closed or semi-closed settings such as dormitories, military centers, resorts, cruise ships, prisons, daycare centers, LTCFs, and hospitals. Norovirus causes substantial societal burden in terms of morbidity and healthcare utilization across the globe. The lack of standard testing for norovirus outside of outbreak settings makes estimation of the true burden of disease challenging from routine sources and is an area for improvement.

There is currently no licensed vaccine for norovirus AGE, and many features of the virus and the human immune response to it have made vaccine development a challenge. Genotype GII.4 is the primary cause of global norovirus AGE; however, non-GII.4 genotypes may be underestimated in existing outbreak-focused surveillance systems. Multivalent vaccines are needed to provide broad coverage of the at-risk population, and regular composition updates based on norovirus epidemiology may be required. Given the substantial morbidity and mortality associated with norovirus AGE, a vaccine to prevent the disease would have a significant impact on global public health.

## Data Availability

The data summarized in this review are from published articles and are publicly available.

## References

[1] Burke, R. M. & Hall, A. J. in *Norovirus* Vol. 1 (ed Melhem, N. M.) Ch. 1–29 (Springer Nature Switzerland AG, 2021).

[2] Institute for Health Metrics and Evaluation (IHME). *GBD Results. Seattle, W. I., University of Washington, 2020*. https://vizhub.healthdata.org/gbd-results/ (2023).

[3] Kirk MD (2015). World health organization estimates of the global and regional disease burden of 22 foodborne bacterial, protozoal, and viral diseases, 2010: a data synthesis. PLoS Med..

[4] Lopman BA, Steele D, Kirkwood CD, Parashar UD (2016). The vast and varied global burden of norovirus: prospects for prevention and control. PLoS Med..

[5] Pires SM (2015). Aetiology-specific estimates of the global and regional incidence and mortality of diarrhoeal diseases commonly transmitted through food. PloS One.

[6] Zhang, X. et al. Global burden and trends of norovirus-associated diseases from 1990 to 2019: an observational trend study. *Front. Public Health* **10** (2022). 10.3389/fpubh.2022.905172 PMC9247406 35784210

[7] Hall AJ (2013). Norovirus disease in the United States. Emerg. Infect. Dis..

[8] Trivedi TK (2013). Clinical characteristics of norovirus-associated deaths: a systematic literature review. Am. J. Infect. Control.

[9] Inns T, Harris J, Vivancos R, Iturriza-Gomara M, O’Brien S (2017). Community-based surveillance of norovirus disease: a systematic review. BMC Infect. Dis..

[10] Grytdal SP (2016). Incidence of norovirus and other viral pathogens that cause acute gastroenteritis (AGE) among Kaiser permanente member populations in the United States, 2012–2013. PLoS One.

[11] Johnston CP (2007). Outbreak management and implications of a nosocomial norovirus outbreak. Clin. Infect. Dis..

[12] Harris JP, Lopman BA, O’Brien SJ (2010). Infection control measures for norovirus: a systematic review of outbreaks in semi-enclosed settings. J. Hosp. Infect..

[13] Matthews JE (2012). The epidemiology of published norovirus outbreaks: a review of risk factors associated with attack rate and genogroup. Epidemiol. Infect..

[14] Adams C (2020). Quantifying the roles of vomiting, diarrhea, and residents vs. staff in norovirus transmission in U.S. nursing home outbreaks. PLoS Comput. Biol..

[15] Mattner F, Mattner L, Borck HU, Gastmeier P (2005). Evaluation of the impact of the source (patient versus staff) on nosocomial norovirus outbreak severity. Infect. Control. Hosp. Epidemiol..

[16] Lopman BA (2004). Epidemiology and cost of nosocomial gastroenteritis, avon, england, 2002–2003. Emerg. Infect. Dis..

[17] MacCannell T (2011). Guideline for the prevention and control of norovirus gastroenteritis outbreaks in healthcare settings. Infect. Control Hospital Epidemiol..

[18] Cardemil CV, Parashar UD, Hall AJ (2017). Norovirus infection in older adults: epidemiology, risk factors, and opportunities for prevention and control. Infect. Dis. Clin. North Am..

[19] Chen Y, Hall AJ, Kirk MD (2017). Norovirus disease in older adults living in long-term care facilities: strategies for management. Curr. Geriatr. Rep..

[20] Rajagopalan S, Yoshikawa TT (2016). Norovirus infections in long-term care facilities. J. Am. Geriatr. Soc..

[21] Lindsay L, Wolter J, De Coster I, Van Damme P, Verstraeten T (2015). A decade of norovirus disease risk among older adults in upper-middle and high income countries: a systematic review. BMC Infect. Dis..

[22] US Centers for Disease Prevention and Control. *Common Settings of Norovirus Outbreaks*. https://www.cdc.gov/norovirus/outbreaks/common-settings.html (2023).

[23] Vega E (2014). Genotypic and epidemiologic trends of norovirus outbreaks in the United States, 2009 to 2013. J. Clin. Microbiol..

[24] Hall AJ (2014). Vital signs: foodborne norovirus outbreaks—United States, 2009–2012. MMWR Morb. Mortal. Wkly Rep..

[25] US Centers for Disease Control and Prevention. *Outbreak Updates for International Cruise Ships: Vessel Sanitation Program 2011* (CDC, 2011).

[26] Jenkins KA, Vaughan GH, Rodriguez LO, Freeland A (2021). Acute gastroenteritis on cruise ships—maritime illness database and reporting system, United States, 2006–2019. MMWR Surveill. Summ..

[27] US Centers for Disease Control and Prevention. *Norovirus Worldwide.* https://www.cdc.gov/norovirus/trends-outbreaks/worldwide.html (2024).

[28] Bartsch SM, Lopman BA, Ozawa S, Hall AJ, Lee BY (2016). Global economic burden of norovirus gastroenteritis. PloS One.

[29] Ahn, S. Y. et al. Changes in the occurrence of gastrointestinal infections after COVID-19 in Korea. *J. Korean Med. Sci.* **36**, e180 (2021). 10.3346/jkms.2021.36.e180 PMC8216988 34155841

[30] Eigner U, Verstraeten T, Weil J (2021). Decrease in norovirus infections in Germany following COVID-19 containment measures. J. Infect..

[31] Mack D (2021). Where have the enteric viruses gone?-differential effects on frequent causes of infectious diarrhoea by SARS-CoV-2 pandemic lockdown measures. Infect. Prev. Pract..

[32] Ondrikova N (2021). Differential impact of the COVID-19 pandemic on laboratory reporting of norovirus and campylobacter in England: a modelling approach. PLoS One.

[33] Kambhampati AK (2022). Notes from the field: norovirus outbreaks reported through NoroSTAT—12 states, August 2012–July 2022. MMWR Morb. Mortal. Wkly Rep..

[34] Atmar RL (2010). Noroviruses: state of the art. Food Environ. Virol..

[35] Green, K. Y. in *Fields Virology 6th* edn, Vol. 1 (ed Knife, D. M.) Ch. 20 (Lippincott Williams and Wilkins, 2013).

[36] McFadden N (2011). Norovirus regulation of the innate immune response and apoptosis occurs via the product of the alternative open reading frame 4. PLoS Pathog.

[37] Parra GI (2019). Emergence of norovirus strains: a tale of two genes. Virus Evol..

[38] Vinjé J (2015). Advances in laboratory methods for detection and typing of norovirus. J. Clin. Microbiol..

[39] Chhabra P (2019). Updated classification of norovirus genogroups and genotypes. J. Gen. Virol..

[40] Winder N, Gohar S, Muthana M (2022). Norovirus: An overview of virology and preventative measures. Viruses.

[41] Mahar JE, Bok K, Green KY, Kirkwood CD (2013). The importance of intergenic recombination in norovirus GII.3 evolution. J. Virol.

[42] Mans J (2016). Norovirus diversity in children with gastroenteritis in South Africa from 2009 to 2013: GII.4 variants and recombinant strains predominate. Epidemiol. Infect..

[43] Medici MC (2014). Novel recombinant GII.P16_GII.13 and GII.P16_GII.3 norovirus strains in Italy. Virus Res..

[44] Hoa Tran TN, Trainor E, Nakagomi T, Cunliffe NA, Nakagomi O (2013). Molecular epidemiology of noroviruses associated with acute sporadic gastroenteritis in children: global distribution of genogroups, genotypes and GII.4 variants. J. Clin. Virol..

[45] van Beek J (2018). Molecular surveillance of norovirus, 2005-16: an epidemiological analysis of data collected from the NoroNet network. Lancet Infect. Dis..

[46] Green KY (2002). A predominant role for Norwalk-like viruses as agents of epidemic gastroenteritis in Maryland nursing homes for the elderly. J. Infect. Dis..

[47] Vinjé J, Koopmans MP (1996). Molecular detection and epidemiology of small round-structured viruses in outbreaks of gastroenteritis in the Netherlands. J. Infect. Dis..

[48] Parra GI (2017). Static and evolving norovirus genotypes: implications for epidemiology and immunity. PLoS Pathogens.

[49] Bull RA, White PA (2011). Mechanisms of GII.4 norovirus evolution. Trends Microbiol..

[50] Ruis C (2017). The emerging GII.P16-GII.4 Sydney 2012 norovirus lineage is circulating worldwide, arose by late-2014 and contains polymerase changes that may increase virus transmission. PLoS One.

[51] Lindesmith LC (2022). Immune imprinting drives human norovirus potential for global spread. Mbio.

[52] Cannon JL (2017). Genetic and epidemiologic trends of norovirus outbreaks in the United States from 2013 to 2016 demonstrated emergence of novel GII.4 recombinant viruses. J. Clin. Microbiol..

[53] van Beek J (2018). Molecular surveillance of norovirus, 2005–16: an epidemiological analysis of data collected from the NoroNet network. Lancet Infect. Dis..

[54] Hoa Tran TN, Trainor E, Nakagomi T, Cunliffe NA, Nakagomi O (2013). Molecular epidemiology of noroviruses associated with acute sporadic gastroenteritis in children: global distribution of genogroups, genotypes and GII. 4 variants. J. Clin. Virol..

[55] Saito M (2014). Multiple norovirus infections in a birth cohort in a Peruvian Periurban community. Clin. Infect. Dis..

[56] NoroSurv. *A Global Network for Norovirus Strain Surveillance Among Children*, 2023 https://www.norosurv.org/login (2023).

[57] Kambhampati AK (2023). Spatiotemporal trends in norovirus outbreaks in the United States, 2009–2019. Clin. Infect. Dis..

[58] Jin M (2020). Norovirus outbreak surveillance, China, 2016–2018. Emerg. Infect. Dis..

[59] Ao Y (2017). Norovirus GII.P16/GII.2-associated gastroenteritis, China, 2016. Emerg. Infect. Dis..

[60] Niendorf, S. et al. Steep rise in norovirus cases and emergence of a new recombinant strain GII.P16-GII.2, Germany, winter 2016. *Euro Surveill*. 10.2807/1560-7917.ES.2017.22.4.30447 (2017). 10.2807/1560-7917.ES.2017.22.4.30447 PMC5388089 28181902

[61] Motoya T (2019). Variation of human norovirus GII genotypes detected in Ibaraki, Japan, during 2012–2018. Gut. Pathog..

[62] Leshem E (2013). Effects and clinical significance of GII.4 Sydney norovirus, United States, 2012–2013. Emerg. Infect. Dis..

[63] Karst SM, Baric RS (2015). What is the reservoir of emergent human norovirus strains?. J. Virol..

[64] Mans, J. Norovirus infections and disease in lower-middle and low-income countries, 1997–2018. *Viruses*10.3390/v11040341 (2019). 10.3390/v11040341 PMC6521228 30974898

[65] Tohma K (2021). Viral intra-host evolution in immunocompetent children contributes to human norovirus diversification at the global scale. Emerg Microbes Infect..

[66] Bhavanam S (2020). Differences in illness severity among circulating norovirus genotypes in a large pediatric cohort with acute gastroenteritis. Microorganisms.

[67] Desai R (2012). Severe outcomes are associated with genogroup 2 genotype 4 norovirus outbreaks: a systematic literature review. Clin. Infect. Dis..

[68] Burke RM (2019). The norovirus epidemiologic triad: predictors of severe outcomes in US norovirus outbreaks, 2009–2016. J. Infect. Dis..

[69] Haddadin Z (2021). Characteristics of GII. 4 norovirus versus other genotypes in sporadic pediatric infections in Davidson County, Tennessee, USA. Clin. Infect. Dis..

[70] Huhti L (2011). Norovirus GII-4 causes a more severe gastroenteritis than other noroviruses in young children. J. Infect. Dis..

[71] Friesema I (2009). Differences in clinical presentation between norovirus genotypes in nursing homes. J. Clin. Virol..

[72] Donaldson EF, Lindesmith LC, Lobue AD, Baric RS (2008). Norovirus pathogenesis: mechanisms of persistence and immune evasion in human populations. Immunol. Rev..

[73] Lindesmith LC (2008). Mechanisms of GII. 4 norovirus persistence in human populations. PLoS Med..

[74] Shioda, K. et al. In *Open Forum Infectious Diseases*. ofx131 (Oxford University Press US, 2021).

[75] Shah MP (2017). Near real-time surveillance of US norovirus outbreaks by the norovirus sentinel testing and tracking network—United States, August 2009–July 2015. Morbid. Mortality Weekly Rep..

[76] Wyatt RG (1974). Comparison of three agents of acute infectious nonbacterial gastroenteritis by cross-challenge in volunteers. J. Infect. Dis..

[77] Blazevic V (2015). Multiple consecutive norovirus infections in the first 2 years of life. Eur. J. Pediatr..

[78] Karangwa CK (2017). Sequential gastroenteritis outbreaks in a single year caused by norovirus genotypes GII.2 and GII.6 in an institutional setting. Open Forum. Infect. Dis..

[79] Parra GI, Green KY (2014). Sequential gastroenteritis episodes caused by 2 norovirus genotypes. Emerg. Infect. Dis..

[80] Chhabra P (2021). Homotypic and heterotypic protection and risk of reinfection following natural norovirus infection in a highly endemic setting. Clin. Infect. Dis..

[81] Sherwood J (2020). Efficacy of an intramuscular bivalent norovirus GI.1/GII.4 virus-like particle vaccine candidate in healthy US adults. Vaccine.

[82] Teunis PF (2008). Norwalk virus: how infectious is it?. J. Med. Virol.

[83] Atmar RL (2014). Determination of the 50% human infectious dose for Norwalk virus. J. Infect. Dis..

[84] de Graaf M, Villabruna N, Koopmans MP (2017). Capturing norovirus transmission. Curr. Opin. Virol..

[85] Kirby AE, Streby A, Moe CL (2016). Vomiting as a symptom and transmission risk in norovirus illness: evidence from human challenge studies. PLoS One.

[86] Chen, Y. et al. Factors driving norovirus transmission in long-term care facilities: a case-level analysis of 107 outbreaks. *Epidemics* **42**, 100671 (2023). 10.1016/j.epidem.2023.100671 PMC11389824 36682288

[87] Kirby AE, Shi J, Montes J, Lichtenstein M, Moe CL (2014). Disease course and viral shedding in experimental Norwalk virus and Snow Mountain virus infection. J. Med. Virol..

[88] Atmar RL (2008). Norwalk virus shedding after experimental human infection. Emerg. Infect. Dis..

[89] Bok K (2016). Epidemiology of norovirus infection among immunocompromised patients at a tertiary care research hospital, 2010–2013. Open Forum. Infect. Dis.

[90] Davis, A. et al. Infectious norovirus is chronically shed by immunocompromised pediatric hosts. *Viruses*10.3390/v12060619 (2020). 10.3390/v12060619 PMC7354526 32516960

[91] Bok K, Green KY (2012). Norovirus gastroenteritis in immunocompromised patients. N. Engl. J. Med..

[92] Graziano, V. R., Wei, J. & Wilen, C. B. Norovirus attachment and entry. *Viruses*10.3390/v11060495 (2019). 10.3390/v11060495 PMC6630345 31151248

[93] Lopman B (2012). Environmental transmission of norovirus gastroenteritis. Curr. Opin. Virol..

[94] Lindesmith L (2003). Human susceptibility and resistance to Norwalk virus infection. Nat. Med..

[95] Nordgren, J. & Svensson, L. Genetic susceptibility to human norovirus infection: an update. *Viruses*10.3390/v11030226 (2019) 10.3390/v11030226 PMC6466115 30845670

[96] Lopman BA (2015). Norovirus infection and disease in an Ecuadorian birth cohort: association of certain norovirus genotypes with host FUT2 secretor status. J. Infect. Dis..

[97] Arrouzet CJ (2020). Population-level human secretor status is associated with genogroup 2 type 4 norovirus predominance. J. Infect. Dis..

[98] Carlsson B (2009). The G428A nonsense mutation in FUT2 provides strong but not absolute protection against symptomatic GII.4 Norovirus infection. PLoS One.

[99] Yen C (2011). Impact of an emergent norovirus variant in 2009 on norovirus outbreak activity in the United States. Clin. Infect. Dis..

[100] Cardoso MR, Cousens SN, de Góes Siqueira LF, Alves FM, D’Angelo LA (2004). Crowding: risk factor or protective factor for lower respiratory disease in young children?. BMC Public Health.

[101] Rohayem J (2009). Norovirus seasonality and the potential impact of climate change. Clin. Microbiol. Infect..

[102] Gruber JF (2017). Risk factors for norovirus gastroenteritis among Nicaraguan children. Am. J. Trop. Med. Hyg..

[103] Wu CY (2021). Clinical characteristics and risk factors for children with norovirus gastroenteritis in Taiwan. J. Microbiol. Immunol. Infect..

[104] Mathew, S. et al. Epidemiological, molecular, and clinical features of norovirus infections among pediatric patients in Qatar. *Viruses*10.3390/v11050400 (2019). 10.3390/v11050400 PMC6563317 31035642

[105] Robilotti E, Deresinski S, Pinsky BA (2015). Norovirus. Clin. Microbiol. Rev..

[106] Alfano-Sobsey E (2012). Norovirus outbreak associated with undercooked oysters and secondary household transmission. Epidemiol. Infect..

[107] Hardstaff JL (2018). Foodborne and food-handler norovirus outbreaks: a systematic review. Foodborne Pathog. Dis..

[108] Kim HY, Kwak IS, Hwang IG, Ko G (2008). Optimization of methods for detecting norovirus on various fruit. J. Virol. Methods.

[109] Lowther JA, Gustar NE, Powell AL, Hartnell RE, Lees DN (2012). Two-year systematic study to assess norovirus contamination in oysters from commercial harvesting areas in the United Kingdom. Appl. Environ. Microbiol..

[110] Tian P, Yang D, Mandrell R (2011). A simple method to recover norovirus from fresh produce with large sample size by using histo-blood group antigen-conjugated to magnetic beads in a recirculating affinity magnetic separation system (RCAMS). Int. J. Food Microbiol..

[111] Kukkula M, Maunula L, Silvennoinen E, von Bonsdorff CH (1999). Outbreak of viral gastroenteritis due to drinking water contaminated by Norwalk-like viruses. J. Infect. Dis..

[112] Johnson JA, Parra GI, Levenson EA, Green KY (2017). A large outbreak of acute gastroenteritis in Shippensburg, Pennsylvania, 1972 revisited: evidence for common source exposure to a recombinant GII.Pg/GII.3 norovirus. Epidemiol. Infect..

[113] Kaplan JE (1982). Epidemiology of Norwalk gastroenteritis and the role of Norwalk virus in outbreaks of acute nonbacterial gastroenteritis. Ann. Intern. Med..

[114] Verhoef L (2015). Norovirus genotype profiles associated with foodborne transmission, 1999–2012. Emerg. Infect. Dis..

[115] Adams C, Peterson SR, Hall AJ, Parashar U, Lopman BA (2022). Associations of infection control measures and norovirus outbreak outcomes in healthcare settings: A systematic review and meta-analysis. Expert. Rev. Anti. Infect. Ther..

[116] Kambhampati A, Koopmans M, Lopman BA (2015). Burden of norovirus in healthcare facilities and strategies for outbreak control. J. Hosp. Infect..

[117] Parrino TA, Schreiber DS, Trier JS, Kapikian AZ, Blacklow NR (1977). Clinical immunity in acute gastroenteritis caused by Norwalk agent. N. Engl. J. Med..

[118] Simmons K, Gambhir M, Leon J, Lopman B (2013). Duration of immunity to norovirus gastroenteritis. Emerg. Infect. Dis..

[119] Dolin R (1972). Biological properties of Norwalk agent of acute infectious nonbacterial gastroenteritis. Proc. Soc. Exp. Biol. Med..

[120] Johnson PC, Mathewson JJ, DuPont HL, Greenberg HB (1990). Multiple-challenge study of host susceptibility to Norwalk gastroenteritis in US adults. J. Infect. Dis..

[121] Rogawski McQuade ET (2020). Protection from natural immunity against enteric infections and etiology-specific diarrhea in a longitudinal birth cohort. J. Infect. Dis..

[122] Atmar RL (2011). Norovirus vaccine against experimental human Norwalk Virus illness. N. Engl. J. Med..

[123] Ramani S, Estes MK, Atmar RL (2016). Correlates of protection against norovirus infection and disease-where are we now, where do we go?. PLoS Pathog..

[124] Hassan E, Baldridge MT (2019). Norovirus encounters in the gut: multifaceted interactions and disease outcomes. Mucosal. Immunol..

[125] Reeck A (2010). Serological correlate of protection against norovirus-induced gastroenteritis. J. Infect. Dis..

[126] Malm M, Uusi-Kerttula H, Vesikari T, Blazevic V (2014). High serum levels of norovirus genotype-specific blocking antibodies correlate with protection from infection in children. J. Infect. Dis..

[127] Lee, R. M., Lessler, J., Lee, R. A., Rudolph, K. E., Reich, N. G., Perl, T. M. & Cummings, D. A. T. Incubation periods of viral gastroenteritis: a systematic review. BMC Infect Dis. 2013;13:446. 10.1186/1471-2334-13-446 PMC3849296 24066865

[128] Lee SG, Cho HG, Paik SY (2015). Molecular epidemiology of norovirus in South Korea. BMB Rep..

[129] Arias C (2010). Epidemiological and clinical features of norovirus gastroenteritis in outbreaks: a population-based study. Clin. Microbiol. Infect..

[130] Lopman BA, Reacher MH, Vipond IB, Sarangi J, Brown DW (2004). Clinical manifestation of norovirus gastroenteritis in health care settings. Clin. Infect. Dis..

[131] Rockx B (2002). Natural history of human calicivirus infection: a prospective cohort study. Clin. Infect. Dis..

[132] Romero C (2017). Incidence of norovirus-associated diarrhea and vomiting disease among children and adults in a community cohort in the Peruvian Amazon basin. Clin. Infect. Dis.

[133] Washington Integrated Food Safety and Center of Excellence. *Norovirus Outbreak Detection and Case Definition*. https://foodsafety.uw.edu/sites/foodsafety.uw.edu/files/documents/norovirus/noro-toolkit-outbreak-detection-and-case-definition.pdf (2023).

[134] Health Protection Surveillance Centre. *Noroviral Infection (Norovirus)*. https://www.hpsc.ie/a-z/gastroenteric/norovirus/casedefinitions/ (2023).

[135] US Centers for Disease Control and Prevention. *Norovirus—Specimen Collection* (U.S. Department of Health & Human Services, 2021).

[136] Costantini V (2010). Diagnostic accuracy and analytical sensitivity of IDEIA Norovirus assay for routine screening of human norovirus. J. Clin. Microbiol..

[137] Jonckheere S (2017). Multicenter evaluation of the revised RIDA® QUICK test (N1402) for rapid detection of norovirus in a diagnostic laboratory setting. Diagn. Microbiol. Infect. Dis..

[138] Kirby A (2010). An evaluation of the RIDASCREEN and IDEIA enzyme immunoassays and the RIDAQUICK immunochromatographic test for the detection of norovirus in faecal specimens. J. Clin. Virol..

[139] US Centers for Disease Control and Prevention. *Responding to Norovirus Outbreaks.* https://www.cdc.gov/norovirus/trends-outbreaks/responding.html (2020).

[140] Chong PP, Atmar RL (2019). Norovirus in health care and implications for the immunocompromised host. Curr. Opin. Infect. Dis..

[141] Netzler NE, Enosi Tuipulotu D, White PA (2019). Norovirus antivirals: where are we now?. Med. Res. Rev..

[142] Kaufman SS, Green KY, Korba BE (2014). Treatment of norovirus infections: moving antivirals from the bench to the bedside. Antivir. Res..

[143] Henke-Gendo C (2009). New real-time PCR detects prolonged norovirus excretion in highly immunosuppressed patients and children. J. Clin. Microbiol..

[144] Ludwig A, Adams O, Laws HJ, Schroten H, Tenenbaum T (2008). Quantitative detection of norovirus excretion in pediatric patients with cancer and prolonged gastroenteritis and shedding of norovirus. J. Med. Virol..

[145] Ford-Siltz LA, Tohma K, Parra GI (2021). Understanding the relationship between norovirus diversity and immunity. Gut. Microb..

[146] Cortes-Penfield NW, Ramani S, Estes MK, Atmar RL (2017). Prospects and challenges in the development of a norovirus vaccine. Clin. Ther..

[147] Tan M (2021). Norovirus vaccines: current clinical development and challenges. Pathogens.

[148] Leroux-Roels I (2022). A randomized, double-blind, placebo-controlled, dose-escalating phase I trial to evaluate safety and immunogenicity of a plant-produced, bivalent, recombinant norovirus-like particle vaccine. Front. Immunol..

[149] ClinicalTrials.gov. *Bethesda (MD): National Library of Medicine (US).* https://clinicaltrials.gov/search?cond=Norovirus&aggFilters=studyType:int&intr=Vaccine&page=2 (2024).

[150] Su W (2015). Production, characterization and immunogenicity of P particles derived from norovirus GII.4 genotype 2004 variant. Acta Virol.

[151] Tamminen K, Lappalainen S, Huhti L, Vesikari T, Blazevic V (2013). Trivalent combination vaccine induces broad heterologous immune responses to norovirus and rotavirus in mice. PLoS One.

[152] Tan M (2008). Noroviral P particle: structure, function and applications in virus-host interaction. Virology.

[153] Atmar RL (2016). Rapid responses to 2 virus-like particle norovirus vaccine candidate formulations in healthy adults: a randomized controlled trial. J. Infect. Dis..

[154] Treanor JJ (2014). A novel intramuscular bivalent norovirus virus-like particle vaccine candidate–reactogenicity, safety, and immunogenicity in a phase 1 trial in healthy adults. J. Infect. Dis..

[155] López, P. et al. Immunogenicity and tolerability of a bivalent virus-like particle norovirus vaccine candidate in children from 6 months up to 4 years of age: A phase 2 randomized, double-blind trial. *Hum. Vaccin. Immunother.*10.1080/21645515.2023.2204787 (2023). 10.1080/21645515.2023.2204787 PMC10294743 37140558

[156] Kim, L. et al. Safety and immunogenicity of an oral tablet norovirus vaccine, a phase I randomized, placebo-controlled trial. *JCI Insight*. 10.1172/jci.insight.121077 (2018). 10.1172/jci.insight.121077 PMC6124525 29997294

